# Refining flowering date enhances sesame yield independently of day-length

**DOI:** 10.1186/s12870-024-05431-8

**Published:** 2024-07-26

**Authors:** Idan Sabag, Shaked Pnini, Gota Morota, Zvi Peleg

**Affiliations:** 1 The Robert H. Smith Institute of Plant Sciences and Genetics in Agriculture, Faculty of Agriculture, Food and Environment, The Hebrew University of Jerusalem, P.O. Box 12, Rehovot, 7610001 Israel; 2 School of Animal Sciences, Virginia Polytechnic Institute and State University, Blacksburg, VA 24061 USA

**Keywords:** Bulk segregation analysis, Genotype $\times$ Environment $\times$ Management Interactions, Photoperiod, Sowing date, Yield components

## Abstract

**Background:**

The transition from vegetative to reproductive growth is a key factor in yield maximization. Sesame (Sesamum *indicum*), an indeterminate short-day oilseed crop, is rapidly being introduced into new cultivation areas. Thus, decoding its flowering mechanism is necessary to facilitate adaptation to environmental conditions. In the current study, we uncover the effect of day-length on flowering and yield components using F$_2$ populations segregating for previously identified quantitative trait loci (Si_DTF QTL) confirming these traits.

**Results:**

Generally, day-length affected all phenotypic traits, with short-day preceding days to flowering and reducing yield components. Interestingly, the average days to flowering required for yield maximization was 50 to 55 days, regardless of day-length. In addition, we found that Si_DTF QTL is more associated with seed-yield and yield components than with days to flowering. A bulk-segregation analysis was applied to identify additional QTL differing in allele frequencies between early and late flowering under both day-length conditions. Candidate genes mining within the identified major QTL intervals revealed two flowering-related genes with different expression levels between the parental lines, indicating their contribution to sesame flowering regulation.

**Conclusions:**

Our findings demonstrate the essential role of flowering date on yield components and will serve as a basis for future sesame breeding.

**Supplementary Information:**

The online version contains supplementary material available at 10.1186/s12870-024-05431-8.

## Background

Optimizing sowing (or planting) dates is a key field management practice for better crop resource utilization and seed-yield maximization. Changing the sowing date (SD) can directly or indirectly affect developmental and growth dynamics through various environmental cues such as day-length (photoperiod), light spectrum intensity, temperature amplitude, and water availability, and their genotype-by-environment interactions [1–3]. Flowering (i.e., the transition from the vegetative to the reproductive phase) is a critical developmental stage that affects final yield. Variation in flowering date can affect the adaptation of crops to specific agro-system conditions [4] and is regulated by a cascade of genes and environmental factors. The association between flowering-related genes and yield components has been demonstrated in various crop-plants [5].

Sesame (*Sesamum indicum* L., 2n = 2x = 26), which belongs to the *Pedaliaceae* family, is an important oilseed crop worldwide. Its seeds are used for various products in the food and pharmaceutical industry. The seeds contain a considerable amount of oil, proteins, and carbohydrates and are rich in essential vitamins and nutrients [6]. Sesame is a short-day erect plant characterized by an indeterminate fluorescence and a stem that is either simple or branching and rigid. The typical growth period ranges from 12 to 16 weeks. The onset of flowering, which marks the transition from the vegetative to the reproductive stage, typically begins about 30 to 40 days after sowing for early varieties, while late varieties may flower about 70 to 80 days after sowing [7, 8]. To date, annual sesame production is  6.8 million tons (2022; https://www.fao.org/faostat/en/#data/QCL), which continues to escalate in response to growing global demand. This growing demand, part of the global trend toward healthier plant-based food sources, opens an opportunity to expand sesame cultivation into new agro-systems and to develop high-yielding varieties with maximum adaptability.

In recent years, advanced genomic resources have been developed for sesame genetic research and breeding [9, 10]. Consequently, numerous quantitative trait loci (QTL) underlying important traits have been discovered through genome-wide association studies [11–13] and segregating bi-parental populations [6]. Alternatively, bulk segregation analysis (BSA) has emerged as a cost-effective and powerful approach for detecting QTL in plants [14, 15]. In sesame, this approach was successfully applied to identify genomic regions regulating male sterility [16], leaf size [17], and seed coat color [18].

For indeterminate crops, the flowering date is a key trait for plant architecture and yield components, as the flowering period continues until plant maturity [19, 20]. In sesame, growing under short-day-length conditions resulted in an eight-day increase in flowering date and a 30% decrease in plant height (PH), resulting in a lower number of capsules [21]. Day-length also affected the lignan content in sesame seeds [22]. Moreover, allelic variation and expression patterns in flowering-related genes are associated with both day-length response and flowering date [23, 24]. Nonetheless, current knowledge of the photoperiod response in sesame remains limited, as does our understanding of its interaction with yield components at both the genomic and field levels. Recently, we discovered a major QTL (Si_DTF QTL) associated with days to flowering (DTF) and seed yield in sesame using genome-wide association mapping under two growing seasons [12]. Here, we developed new segregating populations for this QTL to test its stability under different genetic backgrounds and environmental conditions. The objectives of the current study were to (***i***) phenotypically characterize the F$_2$ population segregating for the Si_DTF QTL under different day-length regimes, (***ii***) validate the previous mapping results under diverse genetic and environmental conditions, and (***iii***) identify additional QTL affecting flowering date. Our findings shed light on the interaction between flowering date and yield components under different day-length conditions and will serve as a basis for future sesame breeding for new agro-systems independent of day-length.

## Methods

### Plant materials

A segregating population was developed through the crossbreeding between S-490 (♀) and S-10 (♂) from the SCHUJI panel [12], characterized by different allelic configurations at the genomic site associated with DTF and seed-yield per plant (SYPP). In previous studies, the genotypes were characterized by early (40) and late (71) DTF for S-490 and S-10, respectively, and by yield performance (14.12 and 6.79 g plant$^{-1}$ for S-490 and S-10, respectively). The progeny of this crossbreeding, the F${_1}$ line, underwent self-pollination to generate the segregating F${_2}$ populations.

### Field experiment and phenotypic characterization

To evaluate the effect of day-length on DTF, morphological traits, and yield components, we conducted a field experiment with two SD at the experimental farm of the Hebrew University of Jerusalem in Rehovot, Israel (34 47 N, 31 54 E, 54 meters above sea level, sandy loam (Rhodoxeralf)). The F${_2}$ population was divided into two SD cycles: the first was sown under optimal conditions and day-length on May 10, 2021 (*n *= 182) and the second on June 14, 2021 (*n *= 187) to mimic a late sowing date. The day-length throughout the growing period for both cycles is presented in Supplemental Figure S1. Both F${_2}$ populations were sown in a complete randomized design in a two-row, 50 cm apart, polyethylene sheet-covered soil bed in 3 plants per meter stand with 3 seeds, which were later cut into one plant. Each parent was sown with 10 replications along the soil bed. The F${_1}$ seeds were only sufficient for the optimal sowing date experiment. Phenotypes were recorded for each individual during the growing season using the Field Book app [25]. DTF was defined as the number of days from sowing to the first open flower. Height to the first capsule (HTFC), nodes to the first capsule (NTFC), and PH were measured at maturity from the soil surface to the first capsule and the plant tip, respectively, with a laboratory measuring tool. The reproductive zone of the main stem (RZ) was calculated as the delta between PH and HTFC, and the reproductive index (RI) was calculated as the ratio between RZ and PH (RZ/PH). Before harvest, branch number per plant (BNPP) and capsule number per plant (CNPP) were counted. At physiological maturity, plants were harvested and sun-dried. The samples were threshed using a laboratory threshing machine (LD 350, WinterSteiger, Reid, Austria). Seeds were counted using a seed counting machine (Data Count S25, Data Technologies) and weighed in analytical lab weight to obtain seed number per plant (SNPP), seed number per capsule (SNPC), thousand-seed weight (TSW), and SYPP.

### Genetic characterization of the Si_DTF QTL

Two weeks after seed germination, young leaf tissue was sampled from each F${_2}$ plant in each cycle, and DNA was extracted using the CTAB method [26]. A genetic marker (SNP 102374:140:+, Supplemental Table S1), which was the most significant for both DTF and SYPP in the Si_DTF QTL interval [12], was developed for screening the populations using high-resolution melt analysis (Thermo Fisher Scientific, Finland). Consequently, it was used to analyze the F${_2}$ populations and served as a representative marker for the QTL. The alleles T and G were denoted by the early (S-490) and late (S-10) flowering parents, respectively, with heterozygotes represented by the allele G/T.

### Bulk-segregation analysis for flowering date

For each F${_2}$ population, two bulks were generated (four bulks in total) by pooling an equal amount of DNA from 20 plants with extreme phenotypes of early or late DTF, respectively. The four bulks, along with the two parental lines, were sent for whole-genome re-sequencing (Macrogene Europe, Netherlands). Library preparation was done using TruSeq DNA PCR Free and 150 base pairs reads length were paired-end sequenced using Illumina NovaSeq 6000 at 30X coverage. Reads were aligned to the sesame reference genome (https://www.ncbi.nlm.nih.gov/assembly/GCF_000512975.1) using Burrows-Wheeler Aligner [27], and variant calling was performed using SAMtools [28]. Gene annotation was performed using SnpEff [29]. The marker file was filtered to include only polymorphic single nucleotide polymorphisms (SNP) that were homozygous. A total of 17,881 and 19,484 SNP remained for further analysis between the bulks at the optimal and late sowing dates, respectively. To identify QTL for DTF between the bulks, we used the $\Delta$SNP-index [30] in the QTLseqr R package [31].

### Expression analysis of candidate genes

To perform expression analysis of the candidate genes discovered in this study, we performed a greenhouse experiment with five plants from each parent sown in a pot under optimal SD conditions. Plants were evaluated for DTF as described above. Leaves from three different plants for each parent were sampled for RNA extraction and qPCR analysis at three time points (36, 38, and 41 days after sowing). RNA isolation was performed using the Plant/Fungi Total RNA Purification Kit (Norgen Biotek, Thorold, Canada) and on-column DNAse treatment (Qiagen, Germantown, MD, USA). First-strand cDNA synthesis was carried out using SuperScript III reverse transcriptase (Invitrogen, USA), and qPCR analysis was carried out using HOT FIREPol EvaGreen qPCR Supermix (Solis BioDyne, Estonia) on the PikoReal RT-PCR system (ThermoFisher Scientific). Gene-specific primers for the candidate genes were designed using primer-BLAST software ([32], Supplemental Table S1). The 2$^{-\Delta \Delta CT}$ method [33] was used to normalize and calibrate transcript values relative to the *Ubiquitin 6* (UBQ6) reference gene (LOC105165183) according to [34].

### Statistical analyses

All the statistical analysis was conducted using JMP Pro 17 (SAS Institute, USA) and R statistical program [35] with a significant level of 5%. A t-test was used to obtain significant differences between the Si_DTF QTL alleles in the populations and the relative expressions between the parents. Analysis of variance (ANOVA) was conducted to obtain the effect of genotype, environment, and their interaction. We evaluated the relationship between traits at each SD using the Pearson coefficient and heatmap using the corrplot package in R [36].

## Results

### Day-length affected all phenotypic traits

**Fig. 1:**
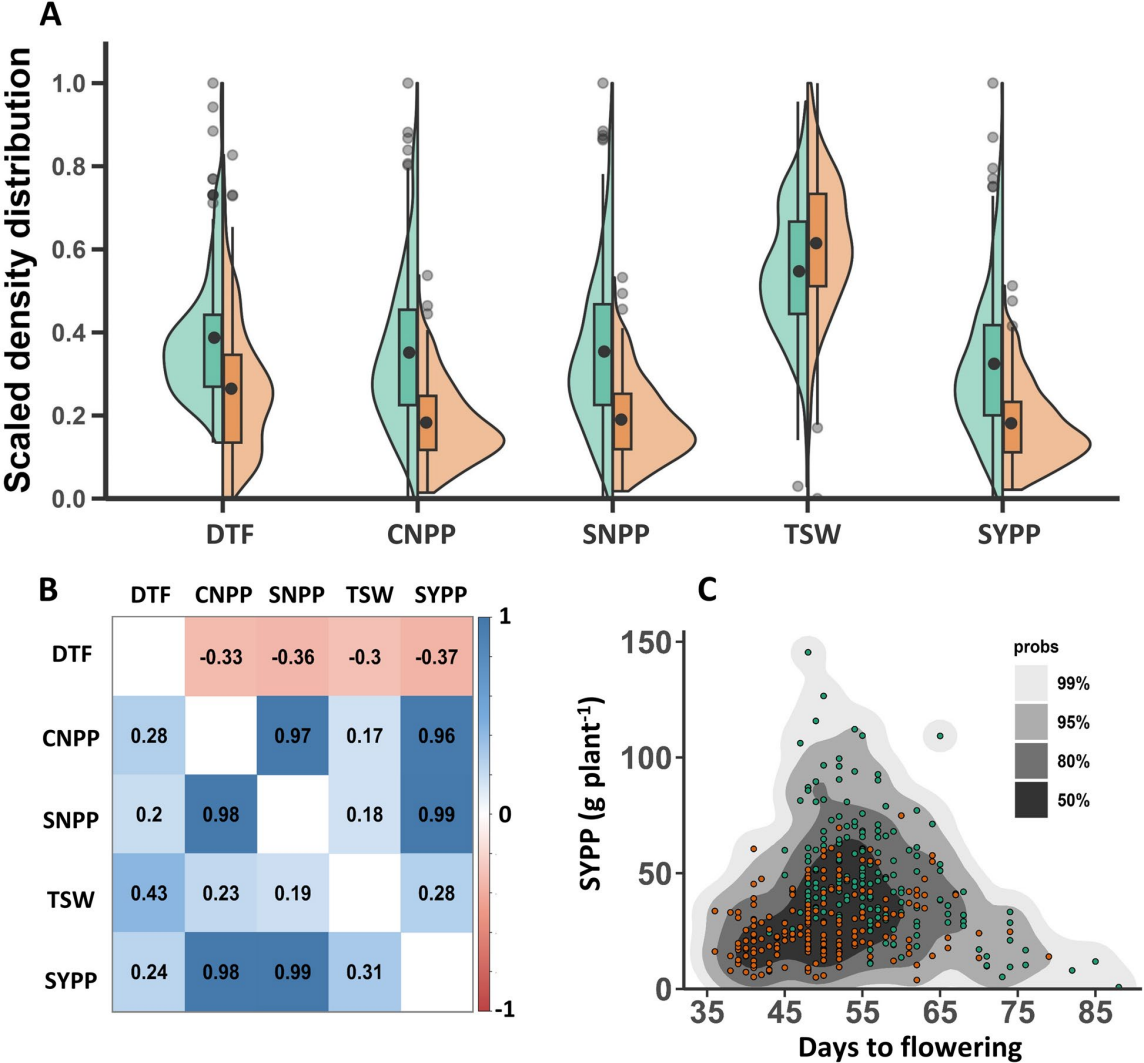
Phenotypic description of flowering and yield components under two sowing dates. **A** Scaled density distribution under optimal (green) and late (orange) sowing dates. Days to flowering (DTF), capsule number per plant (CNPP), seeds number per plant (SNPP), thousand-seeds weight (TSW), and seed-yield per plant (SYPP). **B** Pearson correlation matrix between morphological and yield components under optimal (upper triangle) and late (lower triangle) sowing dates. The colors indicate the degree of correlation from positive (blue) to negative (red). **C** Density plot of the correlation between days to flowering and seed-yield per plant under optimal (green) and late (orange) sowing dates. The colors in the graph range from high (black) to low (light grey) density regions

To assess the phenotypic variation across the two SDs, we conducted a field experiment with F${_2}$ populations (S-490 $\times$ S-10). Overall, we found SD influenced all traits (Fig. 1, Supplemental Tables S2 and S3). DTF ranged from 43 to 88 for optimal SD and 36 to 79 for late SD, with mean values of 56.1 and 49.8 days for optimal and late SD, respectively. Notably, the early flowering parent, S-490, exhibited 44 DTF under optimal SD and 37 DTF under late SD, while the late flowering parent, S-10, flowered at 70 DTF under optimal SD and 74 DTF under late SD (Supplemental Table S2). The morphological traits, HTFC, NTFC, and PH showed lower values under optimal SD (86.6, 10.3, and 185.2 cm, respectively) compared to late SD (105.2, 11.3, and 187.9 cm, respectively). In contrast, RZ and RI values were higher in optimal SD (98.6 cm and 0.53, respectively) than in late SD (82.7 cm and 0.44, respectively). BNPP showed differences in coefficient of variation values, with 35.5 and 47.7 under optimal and late SD, respectively, and mean values of 8.1 *vs*. 5.1 for optimal and late SD, respectively. Yield components varied between SDs (Fig. 1A) when mean CNPP and mean SNPP under optimal conditions were nearly double compared to late SD (282.6 *vs*. 152.5 for CNPP, and 17258 *vs*. 9427 for SNPP, respectively). TSW was higher under late SD (2.84 g) than optimal SD (2.75 g). Interestingly, F${_1}$ lines had higher TSW than both parents under optimal SD (Supplemental Table S2). SYPP showed clear segregation under different SDs. Under optimal SD, SYPP ranged from 0.87 to 145 g plant$^{-1}$ with a mean of 47.8 g plant$^{-1}$, while under late SD, the range narrowed to 3.86 to 74.9 g plant$^{-1}$ with a mean of 27 g plant$^{-1}$.

### Sowing date shapes the flowering and yield components interrelationships

Under optimal SD, DTF showed negative correlations with CNPP (r = -0.33), SNPP (r = -0.36), TSW (r = -0.3), and SYPP (r = -0.37). On the other hand, all these four traits showed positive correlations with DTF under late sowing (r = 0.28, 0.2, 0.43, and 0.24 for CNPP, SNPP, TSW, and SYPP, respectively; Fig. 1B). Examination of the particular relationship between DTF and SYPP under both SDs highlights a concentrated period for yield maximization around 55 DTF, despite the distinct correlations for both traits across different SDs (Fig. 1C). Omitting DTF from the correlation matrix results in positive correlations among all remaining traits, with nearly identical values across different SD (Fig. 1B). All correlations between all measured traits under both SDs are presented in Supplemental Table S4.

### Si_DTF QTL is associated with yield components

**Fig. 2:**
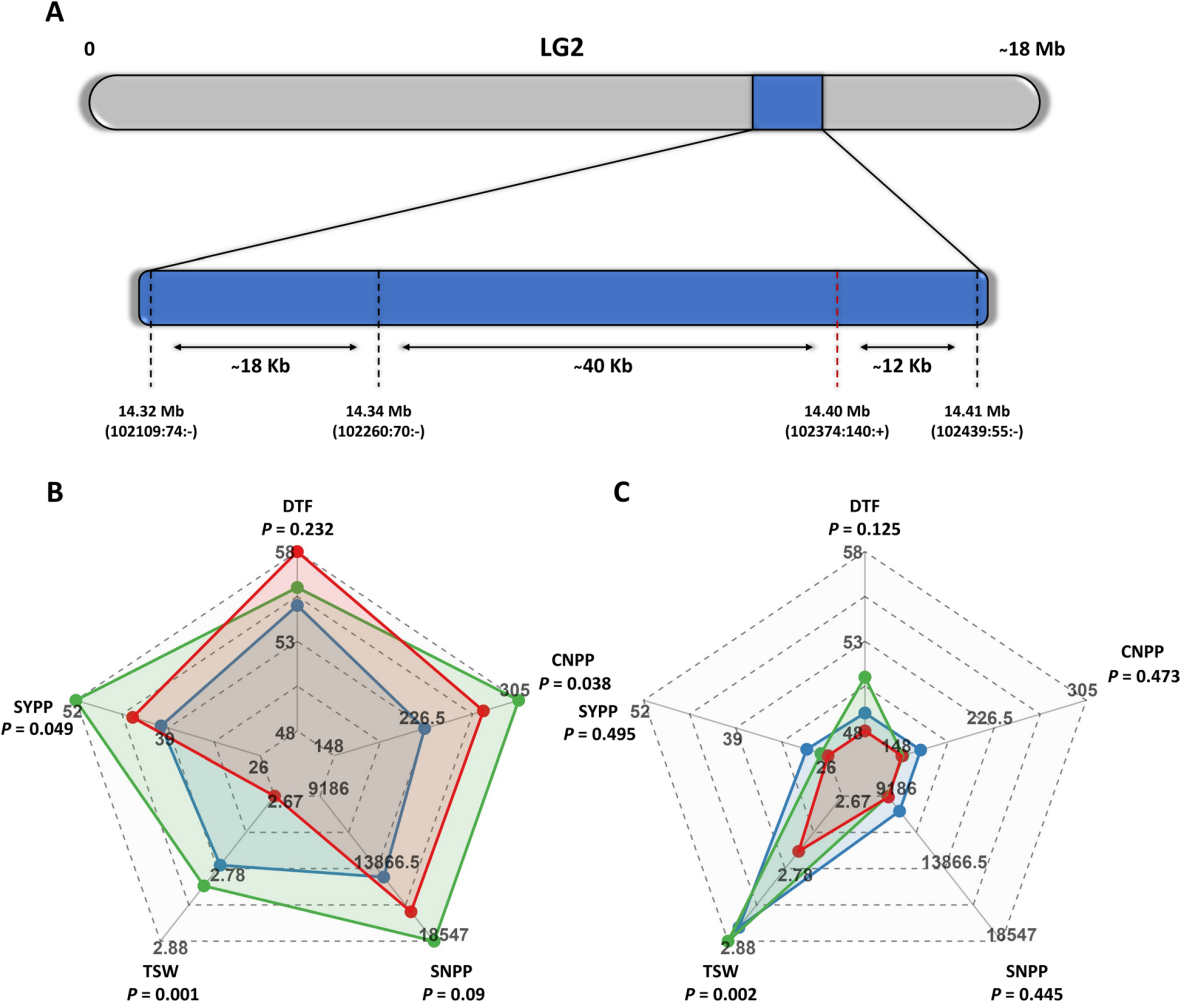
Genetic and phenotypic characterization of Si_DTF QTL. **A** Physical locations and genetic marker intervals of the Si_DTF QTL on linkage group (LG) 2. The red dashed line indicates the marker for the QTL. **B** Radar chart comparing phenology and yield components for each allelic configuration under optimal sowing date. **C** Radar chart comparing phenology and yield components for each allelic configuration under late sowing date. Days to flowering (DTF), capsule number per plant (CNPP), seeds number per plant (SNPP), thousand seeds weight (TSW), and seed-yield per plant (SYPP). Red lines represent homozygous for the T allele (S-490), blue lines represent homozygous for the G allele (S-10), and green lines represent individuals heterozygous for the QTL. The values are ranged between the lowest (center of the radar) and the highest (corner of the radar) average values for each trait and allelic configuration at the two sowing dates

The main goal of the current research was to evaluate the genotype-phenotype relationship and to validate the identified major Si_DTF QTL on linkage group (LG) 2 [12], which was found to be associated with DTF and SYPP (Table S1). Using segregating populations under different SD, we tested the association between allelic configuration and DTF, NCPP, SNPP, TSW, and SYPP (Fig. 2). Under the optimal SD, the allelic configuration was found to be significant for NCPP, TSW, SYPP, and SNPP with *P*-values of 0.038, 0.001, 0.0490, and 0.09, respectively. Interestingly, the heterozygous lines showed the highest values for these traits (Fig. 2B). For DTF, lines with the T allele (inherited from parent S-490) had the highest values, while lines with the G allele (inherited from parent S-10) and heterozygotes had similar values. Under late SD, only TSW showed a significant difference between the different allelic configurations, while the other traits showed no significant variation (Fig. 2C). For DTF and TSW, the highest values were obtained for heterozygous lines (Supplemental Table S4), with a small difference from individuals with the G marker, who had the highest values for CNPP, SNPP, and SYPP traits (Fig. 2C).

**Fig. 3:**
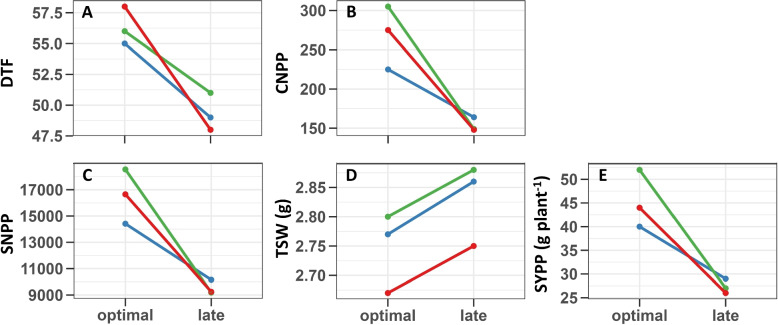
Effect of sowing date on the Si_DTF QTL alleless. Days to flowering (DTF, **A**), capsules number per plant (CNPP, **B**), seeds number per plant (SNPP, **C**), thousand seeds weight (TSW, **D**) and seed-yield per plant (SYPP, **E**). Red lines represent homozygous for the T allele (S-490), blue lines represent homozygous for the G allele (S-10), and green lines represent heterozygous for the QTL

Screening the populations under the two SDs allows us to investigate the interaction between the QTL alleles and SD. Overall, we observed a significant interaction between the QTL and SD for DTF, CNPP, SNPP, and SYPP (Fig. 3 and Table S6). In addition, a similar pattern was observed for DTF, SNPP, and SYPP, where the genetic factor is insignificant while the environmental factor (SD) had a high impact on the traits (Supplemental Table S6). TSW was less affected by the interaction between QTL and SD. However, the statistical analysis showed significant results for the genetic and environmental factors (Fig. 3D and Supplemental Table S6). CNPP was the only yield component significantly influenced by genotype, environment, and their interactions (Fig. 3B and Supplemental Table S6).

### New major QTL conferring days to flowering

**Fig. 4:**
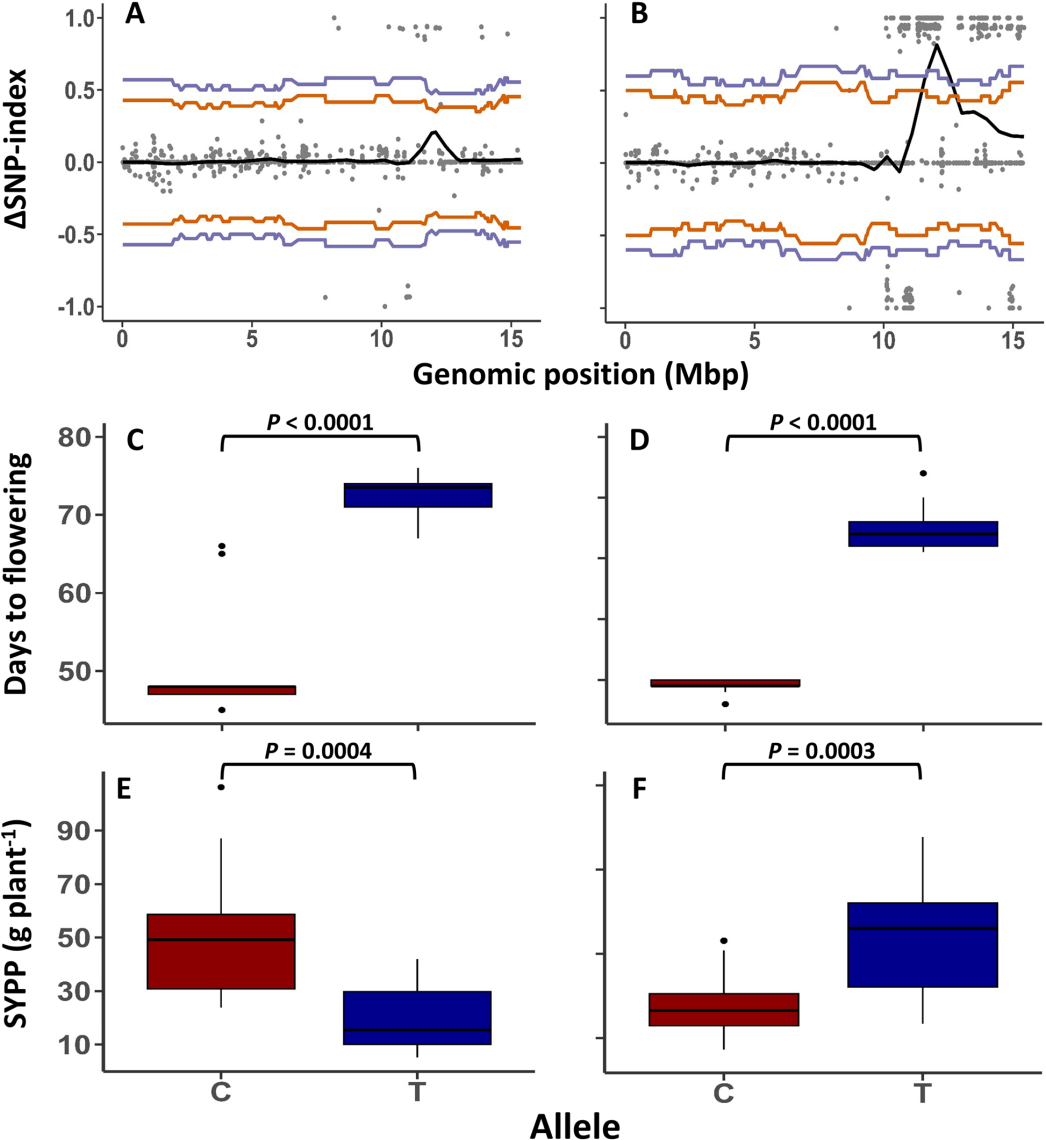
Quantitative trait loci analysis for days to flowering using bulk segregation analysis. The tricube smoothed $\Delta$SNP-index with two confidence intervals at 95% (orange) and 99% (purple) for bulks at optimal (**A**) and late (**B**) sowing dates. Validation of QTL alleles on LG11 for variation in days to flowering and seed-yield under optimal (**C** and **E**) and late (**D** and **F**) sowing dates. C and T represent individuals inheriting the allele from S-490 and S-10, respectively

Following the key role of DTF on yield components (Fig. 1B, C), we conducted a bulk-segregation analysis to uncover additional DTF QTLs by utilizing extreme phenotypes within each F$_2$ population. The mean DTF was 46.57 and 70.3 days for the early and late bulks, respectively, under optimal SD, while under late SD, the means were 38.9 (early) and 65.52 (late). By calculating and smoothing the $\Delta$SNP-index for all markers, we detected a QTL with differential allele frequencies between the bulks on LG11 at both SD (Fig. 4A, B). The QTL spanned a 1.3 Mbp interval, and a scan for candidate genes near its peak revealed two flowering-related genes, *FLOWERING LOCUS T-like* (LOC105174070) and *HEADING DATE 3A* (LOC105174211). Several polymorphisms were identified within these genes between the two parental lines through the re-sequencing process. To validate these results, we scanned bulk individuals for an SNP within *FLOWERING LOCUS T-like* (Fig. 4 and Supplemental Table S7). The C allele (inherited from parent S-490) was found to correlate with early flowering and high yield under optimal SD (Fig. 4C and E), while the T allele (inherited from parent S-10) correlated with late flowering under both SD (Fig. 4C, D). Notably, at late SD, we observed a reverse trend for seed yield, where the T allele was associated with high seed yield, while the C allele was associated with low seed yield (Fig. 4F).

**Fig. 5:**
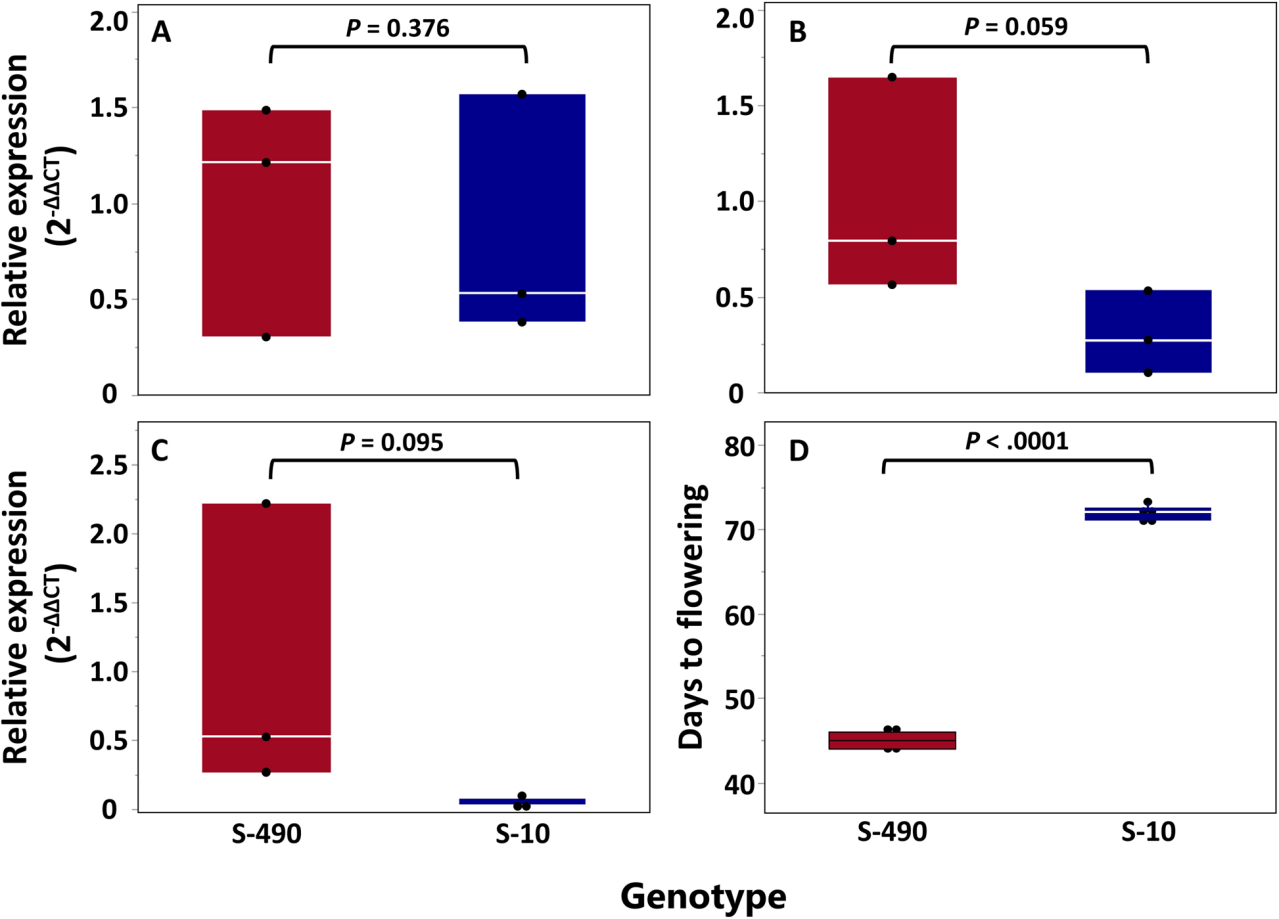
Relative expression of *HEADING DATE 3A* between the parental genotypes. **A** 36, **B** 38, and **C** 41 days after sowing and **D** days to flowering in the greenhouse experiment. *P*-values were determined by t-test, and expression levels were normalized to the UBQ6 reference gene

To further validate these two candidate genes, we characterized their expression pattern along the developmental stages in both parental lines. As these two genes are physically linked, we analyzed the expression of *HEADING DATE 3A*. We sampled and analyzed the transactional pattern of this gene at 36, 38, and 41 days after sowing and measured the DTF of the two parents (Fig. 5). As the parental genotypes (S-490 and S-10) flowered on average at 45 and 71.8 days after sowing, respectively (Fig. 5D), we investigated whether this gene is deferentially expressed between the two parents before flowering induction. After 36 days, the relative expression did not differ between the parents (Fig. 5A), but after 38 and 41 days, we observed a similar trend, where the relative expression was higher for S-490 (Fig. 5B and C), although these differences were below the threshold of significance (*P* $\le$ 0.05).

## Discussion

Adapting to new growing environments is essential for the future food security [37]. Achieving regional adaptation in various crops depends heavily on deciphering the genetic basis of both photoperiod response and flowering time [38–40]. As the transition from vegetative growth to reproductive growth (i.e., flowering) is strongly influenced by day-length, this study focused on characterizing two segregating populations resulting from a cross between an early flowering genotype (S-490) and a late flowering genotype (S-10) under two day-length conditions.

### Sowing date affects sesame development and yield potential

Twelve phenotypes were recorded during the sesame growing season to evaluate the effect of SD on DTF, seed-yield, and yield components (Supplemental Table S3). DTF showed high broad-sense heritability, as previously reported for a sesame diversity panel (SCHUJI, 0.97) [12]. Hence, while DTF showed a similar distribution pattern for both SD with a peak that followed a strong right skew (Fig. 1A), the mean DTF for optimal SD was six days earlier than late SD (Supplemental Table S3), indicating an environmental effect.

Sesame is known as a short-day flowering plant [7], so the shortened day-length was expected to influence DTF. A possible explanation for the decrease in mean DTF among SD can be explained by the fact that the early SD population grew 47 days under extended day-length until June 21 (the northern hemisphere summer solstice), while the late SD population had only 7 days, meaning that it grew entirely under a shorter day-length after germination (Supplemental Figure S1). Similar phenomena have been observed in previous studies, where different SD change the flowering time of sesame [41, 42].

Sesame is characterized by an indeterminate flowering pattern, so optimizing the developmental transition from vegetative growth to the reproductive stage can potentially increase seed-yield by promoting better assimilation allocation for the right balance between growth and yield production. Seed-yield maximization in such crops can be achieved by better regulation of flowering pathways [43–45]. Here, we show that despite the negative and positive correlations between DTF and seed-yield (SYPP) at optimal and late SD, respectively, a yield optimum was set at 50 to 55 DTF (Fig. 1C). This suggests that SD affects overall trait performance but not the optimal DTF needed to maximize seed-yield. Verification of these findings in different environmental conditions and locations could help in the selection of cultivars with optimal DTF, as studied in rapeseed (*Brassica napus* L.) [46].

The morphological traits such as HTFC, NTFC, BNPP, and PH play an important role in the outcome seed-yield (Supplemental Table S4). For example, RI declined by 9% (0.53 *vs*. 0.44 under optimal and late SD, respectively) due to lower HTFC and NTFC under optimal SD and similar PH (Supplemental Table S3). This indicates a more effective reproductive period under optimal SD than late SD, which has also been studied in soybean (*Glycine max* L.) [47]. In addition, BNPP was found to be moderately correlated with SYPP under both SD, but it differed between SDs (8.1 *vs.* 5.1 branches plant$^{-1}$ under optimal and late SD, respectively). Similarly, a previous study showed fewer branches and lower yield under late SD [48]. The early transition to flowering (late SD) leads to fewer branches and capsules, thus reducing the yield potential (Supplemental Tables S3 and S4). Another study showed that delaying SD in sesame results in earlier DTF and reduces the accumulation of dry matter in the stem and leaves, which supports seed-yield [49]. Seed-yield, composed of various yield components [8], was found to be positively and negatively correlated with DTF under optimal and late SD, respectively (Fig. 1B and Table S4). While SNPC and TSW were similar between the SDs, the number of CNPP was strongly affected (282.61 *vs.* 152.52 at optimal and late SD, respectively). As CNPP is correlated with SNPP (Table S4), it serves as the strongest predictor of SYPP (Fig. 1B and Supplemental Table S4).

### Si_DTF is a major QTL affecting yield components

Previously, Sabag et al. [12] identified a significant genomic region on LG2 affecting DTF and SYPP (Si_DTF QTL) in a sesame diversity panel. Here, we validated this QTL (Fig. 2A and Supplemental Table S1) in segregating populations (S-490 $\times$ S-10) under two SDs. ANOVA of optimal SD showed that the allelic configuration has a significant impact on SYPP, whereas, under late SD, neither DTF nor SYPP showed significance (Fig. 2B, C). At optimal SD, we found that this QTL is significantly associated with other yield components such as CNPP, TSW, and SNPP (Fig. 2B), confirming that this QTL is more likely related to seed-yield than to DTF. These yield components also showed compensation, as the T allele (inherited from S-490) promotes high CNPP and SNPP, and the G allele (inherited from S-10) promotes high TSW. Similar compensation between yield components was shown in a previous study in sesame [8]. It is worth noting that our starting point was that two homozygous parents differ in the Si_DTF QTL allele, and therefore, our hypothesis is based on Mendelian segregation in the F$_2$ populations for a ratio of $\frac{1}{4}$ : $\frac{1}{2}$ : $\frac{1}{4}$, but the optimal population exceeded this ratio (Supplemental Table S8), presumably due to the trimming process. Notably, we used two F$_2$ populations that may have unevenly segregated between SDs, so using advanced generation recombinant inbred lines may provide more robust results and additional validation.

At late SD, only TSW was influenced by the presence of the QTL (Fig. 3C), which was in line with the interaction analysis between the QTL and SD performed in this study (Fig. 3 and Supplemental Table S6). Besides TSW, there were interactions between allelic configuration and SD, as T and G/T (heterozygous) configurations were more affected than lines harboring the G allele (Fig. 3A-E and Table S6). When examining the interaction components (Supplemental Table S6), it is clear that SD has a substantial influence, suggesting that environmental factors such as day-length play a major role in these traits, as also shown in the phenotypic analysis (Fig. 1).

TSW had high heritability in sesame compared to other yield components [50, 51], which have advantages and disadvantages of complex traits from a breeding perspective. On the one hand, stability is a promising tool under similar environmental conditions for predicting phenotypes, such as flowering and yield, and incorporating them into new varieties. On the other hand, under variable environmental conditions, the instability of a certain trait may provide flexibility and superior performance to specific environmental conditions but may be more challenging to breed for.

The Si_DTF QTL had a heterotic effect when lines were heterozygous at optimal SD, with an intermediate DTF producing a higher yield (Fig. 2B). These individuals outperformed both homozygous individuals (for either allele) in all yield components, suggesting an overdominance mechanism for these traits. This observation aligns with previous reports indicating that single locus heterosis can improve yield by increasing the number of flowers per plant in tomato (*Solanum lycopersicum*) [52] and by improving yield components in rice (*Oryza sativa*) [53]. Further investigation is required by isolating this QTL and testing its effect on various genetic backgrounds.

### Bulk segregation analysis confirms the phenotypic results

In recent years, BSA has provided an efficient method for detecting QTL by reducing the time-consuming for population development [54]. Here, we exploited the variation in DTF at the two sowing cycles to perform BSA and identify underlying QTL. Using $\Delta$ SNP-index [30], we found major QTL on LG11 for DTF in the two SDs (Fig. 4A, B), which include two homologous flowering-related genes, *FLOWERING LOCUS T-like* and *HEADING DATE 3A*. These two genes were reported in a recent study investigating the major sesame genes regulating flowering [24]. Scanning the bulk individuals for representing SNP within this QTL confirmed our phenotypic correlation between DTF and SYPP under the two SDs (Figs. 2C and 4C-F). Notably, the fact that the BSA analysis did not identify the Si_DTF QTL region further supports that this genomic region is more related to yield than flowering (Fig. 2B).

The pathway of flowering with response to photoperiod involves clock-related genes (i.e., *CONSTANT*) that interact with *FLOWERING LOCUS T-like* to initiate the flowering process [55, 56]. In sesame, *CONSTANT-like* genes are associated with photoperiod response and variation in flowering date [23], which may explain the higher $\Delta$SNP-index values in late SD. As mentioned above, the DTF at late SD was 6 days earlier. As a result, the extreme phenotypes in each cycle also differ when the mean values for the early flowering bulks were 46.57 and 38.9 days, and for the late flowering bulks, the values were 70.3 and 65.52 days for the optimal and late SD, respectively. The reduced day-length in late SD (Supplemental Figure S1) can largely induce *CONSTANT-like* genes and enrich the photoperiod pathway, and as a consequence, larger differences were observed between the allele frequencies at the QTL on LG11, which contains two flowering-related genes in late SD.

The expression analysis of *HEADING DATE 3A* between the parental genotypes showed that there were non-significant differences in the relative expression of this gene near flowering induction, but it was higher in the early genotype (S-490) (Fig. 5A-C). Information from gene annotation analysis reveals that the polymorphism within this gene was located in the 3’ UTR, which mostly affects post-transcriptional mRNA processes such as stability, translation, and localization [57, 58]. These findings can explain the non-significant differences in relative expression and, conversely, the variation in flowering. Since SD only promoted lower DTF for S-490 at late SD (Supplemental Table S2), further studies are needed to determine whether the differences in DTF between the two parental lines are also related to allelic variation and expression in photoperiod-related genes or only to variation in flowering-related genes.

## Conclusion

In this study, we emphasize the importance of day-length on productivity in sesame using segregating populations at two cycles of SD. Our main findings highlight the essential relationship between DTF and seed-yield under different day-length conditions. In addition, we explore this relationship at the genomic level, where we evaluate the effect of two major QTLs (LGs 2 and 11) for these two traits. As we found that refining days to flowering maximizes yield, further studies are needed to decipher the genetic architecture of flowering in sesame to improve this crop’s adaptability to a new agro-system.

### Supplementary Information

Supplementary Material 1.

## Data Availability

No datasets were generated or analysed during the current study.

## Abbreviations

SD: Sowing date
DTF: Days to flowering
SYPP: Seed-yield per plant
QTL: Quantitative trait locus
LG: Linkage group
SNP: Single-nucleotide polymorphism
BSA: Bulk segregation analysis
