# Supplementary material for: Refining flowering date enhances sesame yield independently of day-length
Source: BMC Plant Biol. 2024 Jul 26;24:711. doi: 10.1186/s12870-024-05431-8 (PMC11282604; doi:10.1186/s12870-024-05431-8)
Supplement: Supplementary file 1 — Supplementary Material 1. [file 12870_2024_5431_MOESM1_ESM.pdf]

Refining Flowering Date Enhances Sesame Yield Independently of Day-Length

Idan Sabag[1,2,#], Shaked Pnini[1,#], Gota Morota[2], and Zvi Peleg[1,*]

[1]The Robert H. Smith Institute of Plant Sciences and Genetics in Agriculture, The Hebrew University of Jerusalem, Rehovot 7610001, Israel
[2]School of Animal Sciences, Virginia Polytechnic Institute and State University, Blacksburg, VA 24061, USA

[*] **Corresponding author**: Zvi Peleg,
Robert H. Smith Faculty of Agriculture, Food and Environment,
The Hebrew University of Jerusalem,
P.O. Box 12, Rehovot 7610001, Israel,
Tel: +972-8-9489638
E-mail: zvi.peleg@mail.huji.ac.il
[#] These authors equally contributed to this work

Supplementary materials

Figures

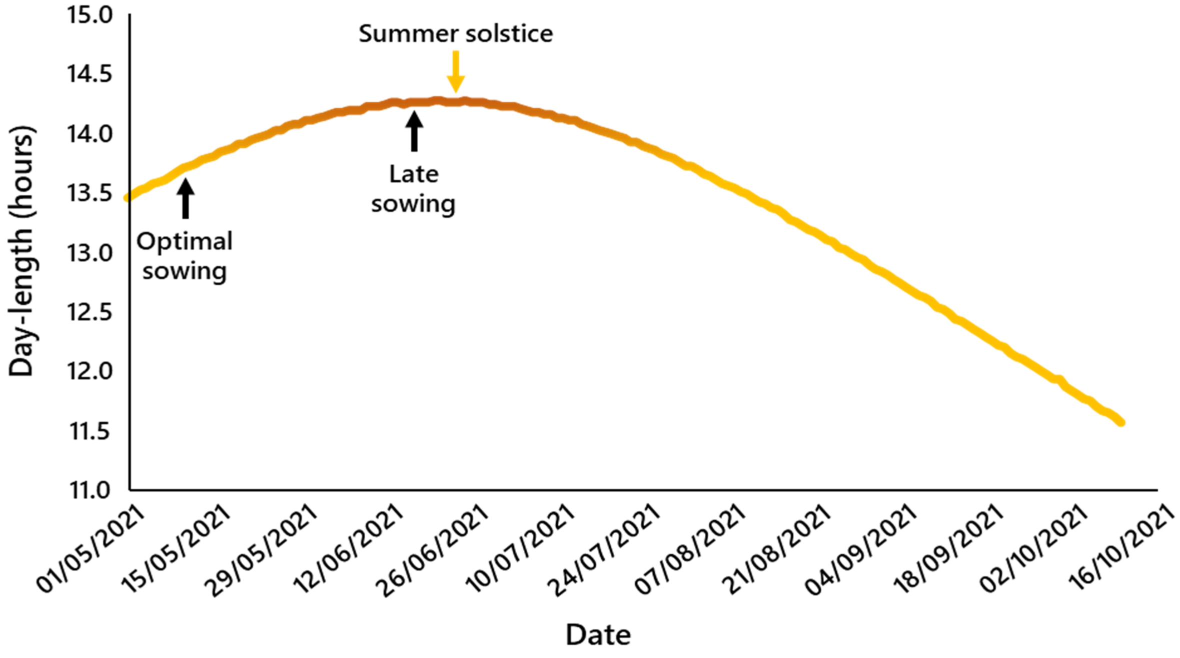

Figure S1: **Day-length duration along the sesame growing season**. The optimal sowing date (10.05.2021), late sowing date (14.06.2021), and the northern hemisphere summer solstice (21.06.2021) are marked on the graph.

Tables

Table S1: **Primers used for genetic characterization of the Si_DTF QTL.** Markers for genotyping using high-resolution melting (HRM) and for candidate gene expression with quantitative PCR (qPCR).

Primer name	Analysis	Sequence	Tm (°C)
Si_FD_HRM_2.4_F	HRM	CACGAACACATGTATACCTACAGG	57
Si_FD_HRM_2.4_R	HRM	GCTGCCGTGATATAGGCTTC	57
Si_HD_2_F	qPCR	GGGAATATCTGCACTGGTTGGT	60
Si_HD_2_R	qPCR	CACGAAAACAAGGCGGTGG	60

Table S2: **Characterization of the parental and their hybrid F_1 lines**. Mean, standard deviation (SD), and coefficient of variation (CV) of the measured traits under optimal and late sowing dates.

**Trait**		**Optimal**			**Late**	
	Parameter	S-10	S-490	F_1	S-10	S-490
**Phenology**						
Days to flowering	Mean	70	44.10	58.63	73.89	37.11
	SD	4.50	4.01	7.11	5.42	1.27
**Morphology**						
Height to first capsule (cm)	Mean	133.67	35.70	88.38	160.33	44
	SD	13.55	10.31	16.27	13.32	13.44
Node to first capsule (nodes)	Mean	18	6.3	10	20.44	5.11
	SD	2.35	1.06	1.85	1.74	1.69
Plant height (cm)	Mean	221.78	112	190.88	231.11	105
	SD	13.96	21.16	18.63	10.24	20.65
Reproductive zone (cm)	Mean	8.11	76.3	102.5	70.78	61
	SD	13.81	20.22	13.07	15.04	13.28
Reproductive index	Mean	0.4	0.68	0.54	0.31	0.59
	SD	0.05	0.1	0.06	0.06	0.08
Branch number per plant	Mean	6.11	6.3	6.25	8.67	5.67
	SD	2.15	3.16	2.38	2.12	3.12
**Yield components**						
Capsule number per plant	Mean	250.78	145.9	223.88	213.56	98.33
	SD	62.31	86.59	122.42	49.95	70.84
Seed number per plant	Mean	15764.67	7396	14799.38	11146.89	5286.11
	SD	4359.32	4827.84	8795.65	3687.09	4020.93
Seed number per capsule	Mean	62.91	49.81	64.79	51.55	53.13
	SD	10.15	5.9	4.13	9.68	6.47
Thousand-seeds weight (g)	Mean	2.75	2.2	2.91	2.86	2.01
	SD	0.13	0.18	0.09	0.24	0.12
Seed-yield per plant (g plant^{-1})	Mean	43.6	16.78	43.25	32.61	10.71
	SD	12.67	12.02	26.33	12.94	8.25

Table S3: **Phenotypic characterization of the F_2 populations.** Mean, standard deviation (SD), and coefficient of variation (CV) of the measured traits under optimal and late sowing dates.

**Trait**	**Optimal**			**Late**		
	Mean	SD	CV(%)	Mean	SD	CV(%)
**Phenology**						
Days to flowering	56.13	7.98	14.23	49.78	8.17	16.41
**Morphology**						
Height to first capsule (cm)	86.56	21.52	24.86	105.18	27.82	26.45
Node to first capsule (nodes)	10.26	2.53	24.69	11.33	3.03	26.74
Plant height (cm)	185.19	26.08	14.08	187.87	35.57	18.93
Reproductive zone (cm)	98.63	20.46	20.74	82.7	19.32	23.36
Reproductive index	0.53	0.09	16.59	0.44	0.082	18.57
Branch number per plant	8.11	2.88	35.48	5.06	2.42	47.7
**Yield components**						
Capsule number per plant	282.61	142.4	50.39	152.52	75.77	49.68
Seed number per plant	17258.62	8995.67	52.12	9427.38	4787.24	50.78
Seed number per capsule	60.56	7.43	12.26	61.73	6.39	10.34
Thousand seeds weight (g)	2.75	0.21	7.73	2.84	0.22	7.81
Seed-yield per plant (g $plant^{-1}$)	47.76	25.66	53.74	26.98	14.37	53.27

Table S4: **Phenotypic correlations (r) and *P*-values between all measured traits.** Upper and lower triangle elements represent optimal and late sowing dates, respectively.

	DTF	PH	HTFC	NTFC	RZ	RI	BNPP	CNPP	SYPP	TSW	SNPP	SNPC
DTF		0.47	0.83	0.76	-0.27	-0.69	-0.18	-0.33	-0.36	-0.30	-0.35	-0.33
		< .0001	< .0001	< .0001	< .0001	< .0001	0.015	< .0001	< .0001	< .0001	< .0001	< .0001
PH	0.64		0.65	0.55	0.59	-0.09	0.12	0.22	0.21	0.03	0.21	-0.09
	< .0001		< .0001	< .0001	< .0001	0.211	0.112	0.002	0.004	0.73	0.005	0.2
HTFC	0.85	0.84		0.89	-0.23	-0.79	-0.07	-0.18	-0.20	-0.14	-0.21	-0.35
	< .0001	< .0001		< .0001	0.001	< .0001	0.38	0.015	0.0054	0.059	0.004	< .0001
NTFC	0.83	0.74	0.88		-0.23	-0.71	-0.01	-0.101	-0.13	-0.22	-0.13	-0.33
	< .0001	< .0001	< .0001		0.002	< .0001	0.38	0.015	0.0054	0.06	0.0039	< .0001
RZ	-0.06	0.63	0.11	0.11		0.71	0.22	0.47	0.48	0.18	0.49	0.25
	0.43	< .0001	0.13	0.15		0.03	< .0001	< .0001	0.01	< .0001	0.0007	0.18
RI	-0.71	-0.25	-0.72	-0.61	0.58		0.17	0.35	0.37	0.15	0.38	0.36
	< .0001	0.0004	< .0001	< .0001	< .0001		0.023	< .0001	< .0001	0.048	< .0001	< .0001
BNPP	0.23	0.37	0.37	0.43	0.15	-0.14		0.69	0.66	0.02	0.68	0.11
	0.001	< .0001	< .0001	< .0001	0.037	0.06		< .0001	< .0001	0.75	< .0001	0.14
CNPP	0.28	0.72	0.50	0.5	0.61	0.03	0.67		0.66	0.02	0.68	0.11
	< .0001	< .0001	< .0001	< .0001	< .0001	0.68	< .0001		< .0001	0.02	< .0001	0.034
SYPP	0.24	0.70	0.46	0.45	0.63	0.06	0.62	0.97		0.29	0.99	0.34
	0.0009	< .0001	< .0001	< .0001	< .0001	0.4	< .0001	< .0001		< .0001	< .0001	< .0001
TSW	0.43	0.45	0.46	0.34	0.17	-0.32	0.01	0.23	0.31		0.177	0.118
	< .0001	< .0001	< .0001	< .0001	0.02	< .0001	0.93	0.002	< .0001		0.01	0.12
SNPP	0.19	0.67	0.42	0.42	0.63	0.098	0.64	0.98	0.99	0.19		0.35
	0.009	< .0001	< .0001	< .0001	< .0001	0.17	< .0001	< .0001	< .0001	0.01		< .0001
SNPC	-0.4	-0.14	-0.31	-0.28	0.18	0.36	-0.05	0.026	0.18	-0.16	0.22	
	< .0001	0.053	< .0001	< .0001	0.01	< .0001	0.51	0.72	0.01	0.03	0.002	

Table S5: Mean, standard deviation (SD), and coefficients of variation (CV) for days to flowering and yield components in F_2 individuals harboring different allelic configurations at the Si_DTF QTL.

**Trait**	**Allele**	**Optimal**			**Late**		
		Mean	SD	CV(%)	Mean	SD	CV(%)
Days to flowering	G	55.0	9.8	17.84	49.2	7.7	15.55
	G/T	55.5	6.3	11.41	50.8	8.5	16.72
	T	57.5	9.1	15.81	47.9	6.9	14.45
Capsule number per plant	G	225.04	119.08	52.91	164.14	80.68	49.15
	G/T	304.72	148.51	48.74	148.92	74.51	50.03
	T	274.90	137.03	49.85	148.02	74.61	50.41
Seed number per plant	G	14413.08	7949.49	55.16	10155.04	4910.02	48.35
	G/T	18546.84	9551.88	51.50	9185.87	4794.20	52.19
	T	16651.41	8328.96	50.02	9243.58	4768.31	51.59
Thousand seeds weight (g)	G	2.76	0.23	8.52	2.86	0.20	7.15
	G/T	2.79	0.21	7.41	2.88	0.20	7.09
	T	2.67	0.18	6.68	2.75	0.22	8.12
Seed-yield per plant (g plant $^{-1}$)	G	40.43	22.66	56.06	29.26	15.05	51.44
	G/T	52.27	27.81	53.22	26.54	14.26	53.75
	T	44.47	22.39	50.34	25.74	14.11	54.83

Table S6: Analysis of variance for the effects of Si_DTF QTL (G), sowing date (E), and their interaction.

**Source of variation**	**DF**	**Mean square**				
		**DTF**	**CNPP**	**SNPP**	**TSW**	**SYPP**
**Si_DTF QTL (G)**	2	30.06<br>0.691	27208<br>0.12	71723397<br>0.25	0.57<br><0.0001	864.24<br>0.13
**Sowing Date (E)**	1	3404.66<br><0.0001	977133.2<br><0.0001	3710525995<br><0.0001	0.54<br>0.0004	25944.77<br><0.0001
**G × E**	2	197.35<br>0.0439	55288.3<br>0.0136	166011862<br>0.0402	0.0012<br>0.9712	1410.084<br>0.0370
**Error**	363	62.61	12704	51245905	0.04	424.13
**Total**	368					

Table S7: Mean, standard deviation (SD), for days to flowering and seed-yield in F_2 bulk individuals harboring different allelic configurations at the QTL on LG11.

**Trait**	**Allele**	**Optimal**		**Late**	
		Mean	SD	Mean	SD
Days to flowering	C	49.6	6.56	39	1.02
	T	72.41	2.87	64.82	3.62
Seed-yield per plant (g plant $^{-1}$)	C	51.6	24.25	18.04	7.94
	T	20.16	12.97	33.65	13.96

Table S8: Chi-squared analysis of allelic segregation among F_2 populations at optimal and late sowing dates.

**Sowing date**	***n***	**Allele**	**Expected**	**Observed**	χ^2	**DF**	***P-value***
Optimal	182	G	45.5	26	8.357	2	0.001
		G/T	91	95	0.176		
		T	45.5	61	5.280		
Late	187	G	46.75	49	0.108	2	0.873
		G/T	93.5	94	0.003		
		T	46.75	44	0.162		
